# Rapid Phenotypic Antibiotic Susceptibility Testing of Uropathogens Using Optical Signal Analysis on the Nanowell Slide

**DOI:** 10.3389/fmicb.2018.01530

**Published:** 2018-07-10

**Authors:** Marta Veses-Garcia, Haris Antypas, Susanne Löffler, Annelie Brauner, Helene Andersson-Svahn, Agneta Richter-Dahlfors

**Affiliations:** ^1^Swedish Medical Nanoscience Center, Department of Neuroscience, Karolinska Institutet, Stockholm, Sweden; ^2^Department of Microbiology, Tumor and Cell Biology, Division of Clinical Microbiology, Karolinska Institutet and Karolinska University Hospital, Stockholm, Sweden; ^3^Division of Proteomics and Nanobiotechnology, Science for Life Laboratory, KTH-Royal Institute of Technology, Stockholm, Sweden

**Keywords:** AST, UTI, antibiotic resistance, diagnostics, microfabrication, algorithm

## Abstract

Achieving fast antimicrobial susceptibility results is a primary goal in the fight against antimicrobial resistance. Standard antibiotic susceptibility testing (AST) takes, however, at least a day from patient sample to susceptibility profile. Here, we developed and clinically validated a rapid phenotypic AST based on a miniaturized nanotiter plate, the nanowell slide, that holds 672 wells in a 500 nl format for bacterial cultivation. The multitude of nanowells allows multiplexing with a panel of six antibiotics relevant for urinary tract infections. Inclusion of seven concentrations per antibiotic plus technical replicates enabled us to determine a precise minimum inhibitory concentration for 70 clinical uropathogenic *Escherichia coli* isolates. By combining optical recordings of bacterial growth with an algorithm for optical signal analysis, we calculated T_lag_, the point of transition from lag to exponential phase, in each nanoculture. Algorithm-assisted analysis determined antibiotic susceptibility as early as 3 h 40 min. In comparison to standard disk diffusion assays, the nanowell AST showed a total categorical agreement of 97.9% with 2.6% major errors and 0% very major errors for all isolate-antibiotic combination tested. Taking advantage of the optical compatibility of the nanowell slide, we performed microscopy to illustrate its potential in defining susceptibility profiles based on bacterial morphotyping. The excellent clinical performance of the nanowell AST, combined with a short detection time, morphotyping, and the very low consumption of reagents clearly show the advantage of this phenotypic AST as a diagnostic tool in a clinical setting.

## Introduction

The emergence and spread of antimicrobial resistant bacteria has become a major global threat to public health. Infections with resistant pathogens lead to significantly increased morbidity and mortality rates and pose a substantial economical challenge on the health care systems (European Centre for Disease Prevention and Control and European Medicines Agency, 2009; Center for Disease Control and Prevention [CDC], 2013; O’Neill, 2016). Effective treatment of bacterial infections requires methods that accurately and quickly identify which antibiotic should be prescribed for each patient. Sub-optimal performances of current technologies for antibiotic susceptibility testing (AST) leave, however, clinicians with no choice but to administer broad-spectrum antibiotics. As this regime contributes to the emergence of resistant strains, novel methods are needed to address this critical step in the diagnostic process.

Numerous molecular methods using genomic markers have been exploited primarily for microbial identification, but also for AST. Molecular ASTs are generally faster than traditional methods as they usually do not require a previous culturing step (Sodowich et al., 2013; Kostić et al., 2015). However, their dependence on the detection of genetic markers restricts molecular ASTs to the current knowledge of resistance mechanisms. Also, the presence of a resistance-associated gene does not necessarily correspond to phenotypic resistance as the latter depends on molecular networks controlling gene expression levels (Giakkoupi et al., 2005; Tato et al., 2010; Olsen et al., 2013). Thus, molecular methods inform only on what treatment a clinician should avoid rather than prescribe.

Conventional methods for phenotypic ASTs remain essential in routine diagnostics as they directly test for bacterial growth on solid or liquid media in the presence of antibiotics. Solid media based ASTs, such as disk diffusion assay or Etest^TM^, require at least 18–22 h for bacteria to grow visibly on agar plates to be able to evaluate growth inhibition with naked eye (Jorgensen and Ferraro, 2009; O’Neill, 2016). Current liquid media based ASTs, such as broth microdilution assays, also require at least 18–22 h of incubation for bacteria to visibly change the turbidity of the culture and enable antibiotic susceptibility determination with naked eye. The turnaround time of these assays can be improved when combined with continuous spectrophotometric monitoring of bacterial growth in automated instruments, showing an average time to AST result of 9–12 h (Sellenriek et al., 2005; Mittman et al., 2009). However, these time frames still constitute a delay to the susceptibility data and a narrow-spectrum antibiotic treatment cannot be initiated until the next day. The arrival of nanotechnology and microfabrication in the field of clinical microbiology has accelerated the appearance of new, rapid phenotypic AST methodologies. Methods based on microfluidic devices, sometimes in combination with imaging algorithms, have been shown to reduce time to result to 6 h or less (Choi et al., 2014; Hou et al., 2014; Li et al., 2014; Puchberger-Enengl et al., 2015; Derzsi et al., 2016; Matsumoto et al., 2016; Baltekin et al., 2017). Most of these methods provide, however, only qualitative susceptibility results and fail to provide quantitative information that defines the minimum inhibitory concentration (MIC). Also, microfluidic-based methods currently on development rarely provide multiplexity, as they test one or two antibiotics instead of a panel of relevant antibiotics that would provide a comprehensive susceptibility profile.

Here, we report the adaptation and clinical validation of the nanowell slide, a miniaturized nanotiter plate for rapid phenotypic AST of UPEC. Holding 672 nanowells with a volume of 500 nl each, the slide can be used to grow bacteria. Using this high-throughput capability, we designed a phenotypic AST targeting uropathogens, the nanowell AST (nwAST), which includes six antibiotics used to treat urinary tract infections (UTIs). We validated the nwAST using 70 clinical uropathogenic *Escherichia coli* (UPEC) isolates. By applying a T_lag_ algorithm (Weibull et al., 2014), phenotypic AST results with MIC values for each antibiotic are rapidly obtained.

## Materials and Methods

### Bacterial Strains, Media, and Antibiotics

Strains included in this study were wild type (wt) *Escherichia coli* laboratory strain W3110 (Karow and Georgopoulos, 1992), BW25113 (Δfnr-771::kan) [National BioResource Project (NIG, Japan):*E. coli*], and the reference strain *E. coli* ATCC 25922 (Oxoid, United Kingdom). The 70 clinical UPEC isolates and one clinical isolate from each of the species *Klebsiella pneumoniae*, *Klebsiella oxytoca*, *Proteus mirabilis*, *Morganella morganii*, *Enterobacter aerogenes*, and *Citrobacter* *koseri* were obtained anonymously from patients with UTIs (Department of Clinical Microbiology, Karolinska University Hospital, Sweden). All UPEC isolates were initially tested with the disk diffusion assay, the routine diagnostic AST performed at the Karolinska University Hospital. Though we aimed to include an equal number of resistant and sensitive isolates to each antibiotic, the low levels of resistance to nitrofurantoin, mecillinam, and cefadroxil prompted us to focus on testing isolates resistant to ampicillin, trimethoprim, and ciprofloxacin. All isolates were stored in glycerol stocks at −80°C. Strains were streaked on Mueller-Hinton II agar plates (Becton Dickinson, United States) and grown at 37°C for 18 h before use. Cation-adjusted Mueller-Hinton II broth (MHII) (Becton Dickinson) was used for growth in liquid media. Antibiotics (Sigma-Aldrich, United States) were prepared in sterile deionized water to generate stock solutions of 25 mg/ml ciprofloxacin hydrochloride (PHR1044), 10 mg/ml cefadroxil (C0650000), 50 mg/ml mecillinam (3447), and 50 mg/ml trimethoprim (46984). DMSO (Sigma-Aldrich) was used to prepare 50 mg/ml nitrofurantoin (46502). Ampicillin was purchased as a ready-made solution of 100 mg/ml (A5354). Stocks were stored at −80°C until use.

### The Nanowell Slide

**FIGURE 1:**
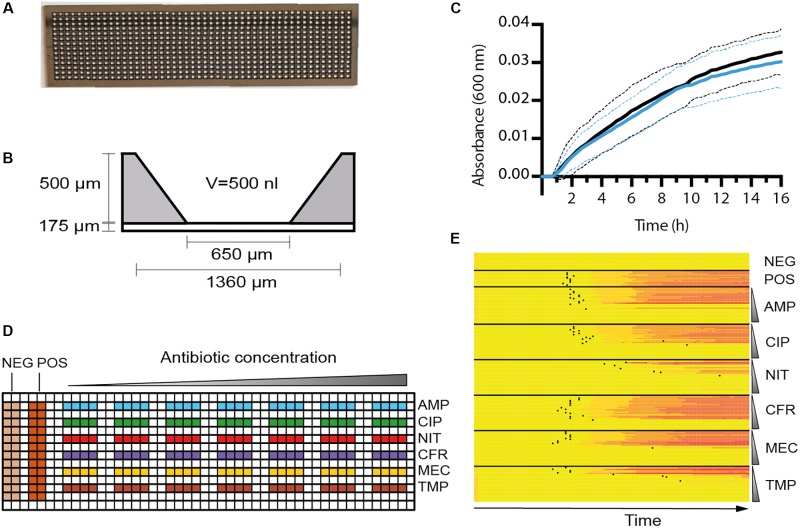
Characterization of the nwSlide as an AST platform. **(A)** Photograph of a nwSlide (25 mm × 75 mm) holding 672 nanowells in a 14 × 48 matrix. **(B)** Side view of one nanowell with dimensions and volume (V) indicated. **(C)** Growth measured at OD_600_ of a wt *Escherichia coli* laboratory strain (W3110, black) and a strain with mutated *fnr* gene (*BW25113Δfnr*, blue) in a nwSlide. Circa 200 nanowells were recorded for each strain, dashed lines = SD, *n* = 3. **(D)** The design of functionalized nwSlides used for nwASTs. The left side offers 24 non-functionalized nanowells each for negative (NEG., medium only) and positive (POS., inoculated medium) controls of bacterial growth. The antibiotics ampicillin (blue, AMP), ciprofloxacin (green, CIP), nitrofurantoin (red, NIT), cefadroxil (purple, CFR), mecillinam (yellow, MEC), and trimethoprim (brown, TMP) are coated and distributed in separate rows. The antibiotic concentration varies from lowest (left) to highest (right) as indicated schematically above the nwSlide. Each antibiotic is represented by seven twofold dilutions. Each concentration includes four nanowells that serve as technical replicates. Antibiotic concentrations in individual experiments are defined in Section “Materials and Methods.” **(E)** MIC determination of the reference strain *E. coli* ATCC 25922 from one nwAST functionalized as in **(D)**. The heatmap shows OD_600_ recordings in each of the 216 nanocultures over 12 h at indicated conditions. A color change from yellow (low OD_600_) to red (high OD_600_) in one row indicates bacterial growth in the corresponding nanowell. Negative and positive controls include 24 wells each. For each antibiotic, growth pattern of the 28 nanocultures at 7 antibiotic concentrations is shown. The vertical gradient symbol indicates that nanocultures in the upper rows are exposed to the lowest concentration of antibiotics, whereas a gradual increase leaves the lower rows representing nanowells exposed to the highest concentration. Black dots represent the T_lag_ of each nanoculture.

The nanowell slide is a glass slide (75 mm × 0.175 mm × 25 mm) bonded to an etched silicon grid that creates a 14 × 48 matrix of 672 nanowells. The outward tilted walls increase the surface area of the nanowells from bottom (650 μm × 650 μm) to top (1,360 μm × 1,360 μm) (**Figure 1A**), resulting in a volume of 500 nl. The slide design and microfabrication are described in Lindström et al. (2008) and Weibull et al. (2014). A sterile, clear polyester adhesive membrane (Thermo Fisher Scientific, United States) cut to the slide’s dimensions was used to seal the nanowells for bacterial culturing. To functionalize the nanowell slide with antibiotics, 500 nl of defined concentrations of antibiotics, representing seven twofold serial dilutions, were pipetted in individual nanowells according to the pattern shown in **Figure 1D**. After loading the antibiotics, the slides were dried at 37°C overnight to passively coat the surface of the nanowells and stored at −20°C until use. Antibiotic concentrations used for the reference strain ATCC 25922 were ampicillin (0.5–32 μg/ml), ciprofloxacin (0.003–0.25 μg/ml), nitrofurantoin (2–128 μg/ml), cefadroxil (2–128 μg/ml), mecillinam (0.03–2 μg/ml), and trimethoprim (0.25–16 μg/ml). For testing of the clinical UPEC strains, ampicillin (0.25–16 μg/ml), ciprofloxacin (0.03–2 μg/ml), nitrofurantoin (2–128 μg/ml), cefadroxil (0.5–32 μg/ml), mecillinam (0.25–16 μg/ml), and trimethoprim (0.125–8 μg/ml) were used.

To enable spectrophotometric recordings, the nanowell slide was placed in a custom microtiter plate adaptor (Weibull et al., 2014) compatible with an Infinite^®^ M1000 PRO plate reader (TECAN, Switzerland) or any other microtiter plate reader. The slide dimensions were added to the plate geometry editor of the Magellan software (version 7.2) (TECAN, Switzerland) in the plate reader to read each nanowell individually.

### Test of Aerobic Growth in the Nanowells

Overnight cultures of wt *E. coli* laboratory strain (W3110) and Δ*fnr-771::kan* (BW25113) in MHII broth were diluted 1:100 in fresh MHII, incubated at 37°C until OD_600_ = 0.4, then further diluted 1:100 in MHII broth. The two strains were each inoculated in half of the nanowells in a slide without any antibiotics. The inoculated nanowell slide was incubated for 16 h in an Infinite^®^ M1000 PRO plate reader. OD_600_ was automatically recorded every 10 min for 16 h at 37°C. Data from each nanowell were blanked by subtracting the average of the first five recordings from the rest of the values of the growth curve.

### Bacterial Inoculation, MIC and T_lag_ Determination in the nwAST

Mueller-Hinton II broth with no bacteria (negative control) was pipetted on columns 1 and 2 at the left side of the slide and wells were covered with the membrane. The bacterial inoculum was prepared by touching 6–8 colonies of each isolate grown on an agar plate, re-suspending them in MHII broth and adjusting turbidity to a 0.5 McFarland standard. The bacterial suspension was further diluted 1:100 to achieve the desired bacterial concentration. This inoculum was pipetted on columns 4–5 (positive control), and 500 μl was then evenly spread over the full slide by lowering the membrane on the slide from left to right. The nwAST was incubated in the plate reader for 12 h at 37°C with a time interval (D_t_) between OD_600_ recordings of 10 min. These OD_600_ recordings were processed to calculate the T_lag_ value. T_lag_ defines the time point when a nanoculture transitions from lag to logarithmic phase, and therefore it also represents the earliest time point when a resistant strain can be differentiated from a susceptible one. To calculate the T_lag_ of each nanoculture we used an algorithm previously developed by our group (Weibull et al., 2014). Briefly, the change in average OD_600_ over a period of time (ΔMOD) is calculated by subtracting the average OD_600_ of seven measurements preceding and including the time point in question (t) from the average OD_600_ of the seven measurements following and including the time point in question:

$$\Delta {\text{MOD}}_{\text{t}}=\frac{\sum_{\text{t}+6}^{\text{t}}\text{OD}}{7}-\frac{\sum_{\text{t}}^{\text{t}-6}\text{OD}}{7}$$

Then the difference between two ΔMOD values of successive time points is calculated (ΔΔMOD). If ΔΔMOD is ≥ 0.001 for six successive time points then the first time point (t) is defined as the T_lag_. Because the calculation of T_lag_ requires the ΔΔMOD of six successive time points, the diagnostic time (T_Dx_) is defined as T_Dx_ = T_lag_ + (12 × D_t_), where D_t_ is the time interval between OD_600_ recordings. The MIC for each antibiotic was then defined as the lowest antibiotic concentration in which no T_lag_, and therefore no bacterial growth, was detected in at least three out of four technical replicates. MIC values were interpreted according to EUCAST guidelines. If a sudden peak or a succession of peaks of absorbance were recorded in a nanowell during incubation, these wells were excluded from the analysis as artifacts.

To plot growth from all nanowells simultaneously, heatmaps were generated in R (version 3.3.2) using the “ggplots” package. Each row in a heatmap represents OD_600_ values over time for one nanoculture. The shift from yellow to red symbolizes the increase in OD_600_ and therefore bacterial growth, a constant yellow row means no bacterial growth.

### Evaluation of Clinical Performance in the nwAST

The AST results obtained for the 70 UPEC strains with the nwAST device were compared to results from the disk diffusion assay (reference method) performed at Karolinska University Hospital. Categorical agreement (CA), major errors (MEs), and very major errors (VMEs) for each antibiotic were calculated in accordance to the FDA guidance for AST systems as shown below:

$$\text{CA}=\frac{\#\;\text{nwAST}\;\text{tests}\;\text{with}\;\text{interpretive}\;\text{agreement}\;\text{to}\;\text{disk}\;\text{diffusion}\;\text{tests}}{\text{Total}\;\#\;\text{of}\;\text{isolates}}\times 100$$

$$\text{ME}=\frac{\#\;\text{susceptible}\;\text{isolates}\;\text{by}\;\text{disk}\;\text{diffusion}\;\text{but}\;\text{resistant}\;\text{by}\;\text{nwAST}}{\text{Total}\;\#\;\text{susceptible}\;\text{isolates}\;\text{by}\;\text{disk}\;\text{diffusion}}\times 100$$

$$\text{VME}=\frac{\#\;\text{resistant}\;\text{isolates}\;\text{by}\;\text{disk}\;\text{diffusion}\;\text{but}\;\text{susceptible}\;\text{by}\;\text{nwAST}}{\text{Total}\;\#\;\text{resistant}\;\text{isolates}\;\text{by}\;\text{disk}\;\text{diffusion}}\times 100$$

Isolates presenting discrepancies between both methods were independently re-tested using the disk diffusion method. Concordance of two results was considered correct.

### Morphotyping of Bacteria in the nwAST

Nanowell ASTs with UPEC clinical isolates ARD311 and ARD318 were incubated at 37°C for 3 h. Since the nanowell slide has the same dimensions as standard microscopy slides, the nwAST was easily fitted on a microscope stage for direct examination of live bacteria in the nanowells under phase contrast microscopy (Nikon TS 100) at 100x magnification. A digital camera (C11440, Hamamatsu) attached to the microscope was used to capture images of bacterial morphotypes, in nanowells containing either the MIC concentration (sensitive isolates) or the breakpoint concentration (resistant isolates) of each antibiotic. Images from the antibiotic-free control were also included. Morphotyping on four collected images per condition was performed with ImageJ v1.46r (Schneider et al., 2012). Image binarization was performed with global thresholding to set bacteria apart from the background. Perimeter, circularity, major and minor axes were calculated for each bacterium.

## Results

### Design and Optimization of the nwAST for Uropathogens

The nanowell slide represents a platform that holds 672 miniaturized nl format (**Figure 1A**). To test whether the nanowell slide can be used as a generic platform for uropathogens other than *E. coli*, we inoculated individual slides with each of the clinical isolates of *Klebsiella* *pneumoniae*, *Klebsiella* *oxytoca*, *Proteus* *mirabilis*, *Morganella* *morganii*, *Enterobacter* *aerogenes*, and *Citrobacter* *koseri*. Each inoculated nanowell slide was placed in a plate adaptor fitted for a plate reader. The growth of 50 randomly selected nanocultures of each strain was monitored spectrophotometrically, while incubating the slide at 37°C. Compilation of growth curves into heatmaps revealed a uniform shift from lag to logarithmic phase for all cultures of respective species (Supplementary Figure S1). This demonstrates a wide usability of the nanowell platform for the most common uropathogens.

As each nanowell harbors the small volume of 500 nl (**Figure 1B**), we next tested whether growth conditions remain aerobic during bacterial cultivation. Using genetically modified bacteria as biosensors, we inoculated 50% of the nanowells in one slide with a wt *E. coli* laboratory strain known to grow both aerobically and anaerobically. In the remaining wells, we applied a strain with a mutation in the *fnr* gene (Δ*fnr*), which is known to dramatically impair growth under anaerobic conditions (Lambden and Guest, 1976). After 16 h incubation performed as described for the uropathogens, analysis of growth curves showed that the wt and the Δ*fnr* mutant strains grew with a similar rate (wt = 0.019 ± 0.003 min^−1^; Δ*fnr* = 0.018 ± 0.003 min^−1^) (**Figure 1C**). This proved that conditions within the nanowells remain aerobic for at least 16 h, which is far longer than the expected time for the AST assay. The nanowell slide is thus well-adapted as a platform for AST of UTI, as it complies with the EUCAST guidelines that recommend aerobic conditions when performing AST on uropathogenic bacteria.

To translate the nanowell slide into a platform for AST in the nanoscale format, we selected a panel of six antibiotics routinely used in the clinics to treat UPEC infections. This included ampicillin, ciprofloxacin, nitrofurantoin, cefadroxil, mecillinam, and trimethoprim. The concentration range of each antibiotic was chosen according to FDA guidance for AST systems (U.S. Department of Health and Human Services et al., 2009), which states that it should span at least two twofold values above and below the MIC breakpoints. We therefore designed the layout of the nwAST to fit seven twofold dilutions for each antibiotic around the EUCAST clinical breakpoints (**Figure 1D**). In contrast to conventional AST methods that rely on just one technical replicate per sample, the numerous wells in our miniaturized device allow inclusion of several ones. We took this opportunity to minimize the risk of false diagnosis by including four technical replicates per concentration. This generates 28 nanocultures for each antibiotic, adding up to 168 nanocultures in total for the six antibiotics present in one nwAST assay. Additionally, we included 24 nanowells each for the negative (only growth media) and positive (growth media and bacteria) controls. In total, this design runs 216 individual bacterial nanocultures of one strain in parallel. To optimize the nwAST workflow we first used the *E. coli* reference strain ATCC 25922 at an inoculum size of 5 × 10^5^ cfu/ml, which corresponds to ≈50 bacteria per nanowell (Supplementary Figure S2). Recording the absorbance of 216 nanowells every 10 min during 12 h incubation generates more than 15,000 data points for one nwAST. Rapid processing of these data is essential to identify the susceptibility profile of bacteria. Thus, we applied the T_lag_ algorithm, which is obtained by processing the absorbance recordings from each of the 216 nanowells. Calculation of T_lag_ establishes the time point when a nanoculture shifts from lag to logarithmic phase and is therefore exclusively associated with growing cultures. We further assisted the visualization of this vast amount of data by plotting in a heatmap, where growth patterns under exposure to different concentrations of each antibiotic are easily observed as gradual shifts from yellow to red in growing cultures (**Figure 1E**). The combination of heatmap representation and T_lag_ determination thus provides a workflow for effortless determination of the MIC for each antibiotic.

### Clinical Validation of nwAST for UPEC Isolates

Once we established an optimized workflow from sample preparation to data analysis we proceeded to clinically validate the nwAST. We collected 70 UPEC strains isolated from patients diagnosed with UTI at the Karolinska University Hospital and analyzed their susceptibility profiles in the nwAST. Results were compared to corresponding results from the disk diffusion assay, which served as a reference method. Based on the evaluation parameters suggested by FDA, we calculated the CA, as well as discrepancies defined as MEs and VMEs, by comparing the number of resistant and sensitive isolates identified with the nwAST and the disk diffusion assay, respectively (**Table 1**). The CA for ampicillin is 95.7%, and the misidentification of three isolates as resistant instead of sensitive resulted in 8.3% ME. Ciprofloxacin has a CA of 98.6% as only one strain was misidentified as resistant (ME 1.6%). For nitrofurantoin and mecillinam, the nwAST identified all isolates correctly resulting in a CA of 100%. Cefadroxil showed a CA of 95.7% as three isolates were misidentified as resistant instead of sensitive (ME 4.3%). Lastly, trimethoprim showed a CA of 97.1% due to misidentification of two isolates as resistant instead of sensitive (ME 4.6%). With CA greater than 95.7% for all antibiotics, we found an excellent CA between the nwAST and the disk diffusion result. This is further substantiated by the very low discrepancies between the nwAST and the disk diffusion. No discrepancies of any type were found between the methods for two of the antibiotics. The remaining four showed a ME of 8.3% or less. Moreover, none of the isolates was misidentified as sensitive instead of resistant, which generated 0% VME for all antibiotics. Taken together the overall CA of the nwAST is 97.9% and the ME 2.6%. Collectively, this experimental analysis verifies the clinical capability of the nwAST to be used as a diagnostic tool.

**Table 1:** Clinical performance of the nwAST compared to the reference disk diffusion method.

Antibiotic	Isolates^1^	Disk diffusion^2^		nwAST^2^		Categorical agreement (%)	Discrepancies (%)	
		R	S	R	S		ME^3^	VME^4^
Ampicillin	70	34	36	37	33	95.7	8.3	0
Ciprofloxacin	70	7	63	8	62	98.6	1.6	0
Nitrofurantoin	70	0	70	0	70	100	0	N/A^5^
Cefadroxil	70	0	70	3	67	95.7	4.3	N/A
Mecillinam	70	1	69	1	69	100	0	0
Trimethoprim	70	26	44	28	42	97.1	4.6	0
**Overall evaluation of the nwAST**						97.9	2.6	0

*^1^Total number of clinical isolates tested. ^2^Number of isolates classified as resistant (R) or sensitive (S) using the disk diffusion or the nwAST methods. ^3^Major errors. ^4^Very major errors. ^5^Not applicable.*

### Rapid Algorithm-Assisted Determination of Antibiotic Susceptibility

**FIGURE 2:**
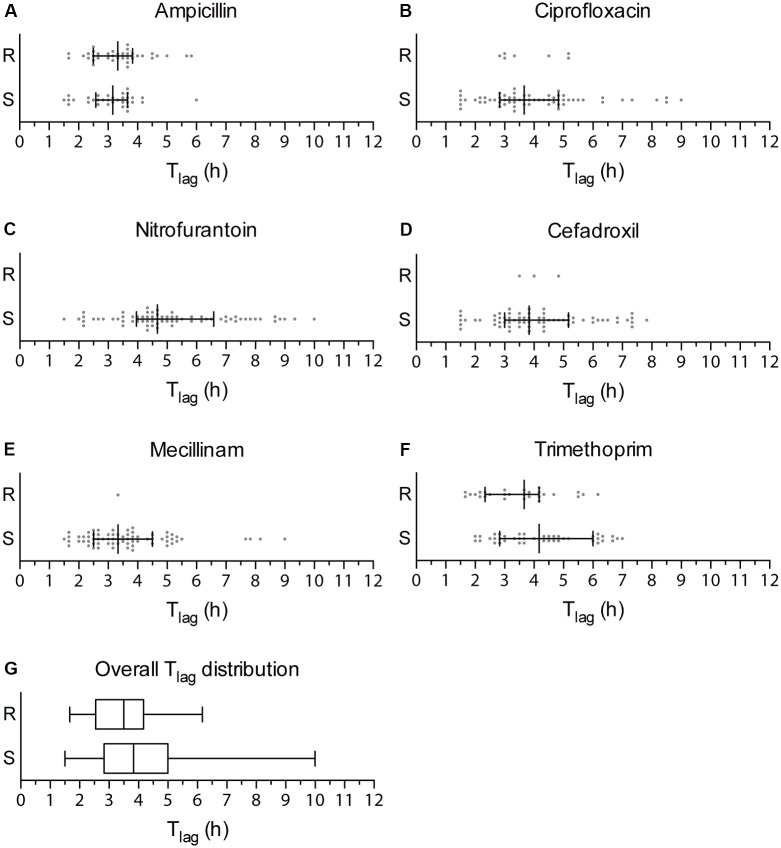
T_lag_ distribution of clinical UPEC isolates. **(A–F)** Dot plots showing the T_lag_ generated by each of the 70 clinical UPEC isolates at the highest antibiotic concentration they were able to grow at. Strains are grouped as resistant strains (R), which generated a T_lag_ above the clinical breakpoint, and susceptible strains (S), which generated a T_lag_ below the clinical breakpoint of each antibiotic. Median is shown with whiskers drawn at the 25^th^ and 75^th^ percentile. **(G)** Box plots showing the overall distribution of T_lag_ values obtained for resistant (R) and sensitive (S) strains irrespective of the type of antibiotic. Whiskers show the minimum and maximum value of T_lag_.

An AST method should provide a fast response to clinicians on which antibiotic is most appropriate to prescribe. The T_lag_ algorithm processes the optical recordings and enables detection of phenotypic resistance at the earliest time possible for an absorbance-based AST. To calculate the T_lag_ of clinical isolates when exposed to antibiotics, we plotted the average T_lag_ obtained from the four technical replicates at the highest concentration of each antibiotic at which isolates were able to grow. The T_lag_ values from isolates were separated in two groups based on their resistance or susceptibility as previously determined (**Figure 2**). To define the shortest time when the nwAST can detect resistance, we focused on T_lag_ obtained above the clinical breakpoint (**Figures 2A–F**). Isolates resistant to ampicillin grew between 1 h 40 min and 5 h 50 min [Median (M) = 3 h 20 min], to ciprofloxacin between 2 h 50 min and 5 h 10 min (M = 3 h 20 min) and to trimethoprim between 1 h 40 min and 6 h 10 min (M = 3 h 40 min). The few resistant isolates to mecillinam and cefadroxil grew as early as 3 h 20 min and 3 h 30 min respectively. No T_lag_ was obtained for nitrofurantoin, as there were no resistant isolates. Taken together, our data showed that the overall T_lag_ distribution had a median of 3 h 30 min for resistant UPEC isolates (**Figure 2G**), with a minimum T_lag_ of 1 h 40 min and a maximum T_lag_ of 6 h 10 min. This reflects the heterogeneity among clinical isolates in relation to the mode of action of individual antibiotics. Taking into account the minimum T_lag_ calculated and 12 additional time points required by the T_lag_ algorithm, the nwAST can detect resistant isolates as early as 3 h 40 min.

The strength of the nwAST is not limited to rapid differentiation between resistant and susceptible strains. Contrary to other fast AST methods, which test bacteria at only one antibiotic concentration above the clinical breakpoint, the large number of nanowells in the nwSlide allows us to test bacteria at five twofold concentrations below that breakpoint. Thus, the nwAST do not only discriminate between susceptible and resistant strains, but also defines a MIC for susceptible isolates, which can only grow and generate a T_lag_ below the clinical breakpoint. Based on the T_lag_ distribution of susceptible isolates (**Figures 2A–F**), they grew within 1 h 30 min–6 h in ampicillin (M = 3 h 10 min), 1 h 30 min–9 h in ciprofloxacin (M = 3 h 40 min), 1 h 30 min–10 h in nitrofurantoin (M = 4 h 40 min), 1 h 30 min–7 h 50 min in cefadroxil (M = 3 h 50 min), 1 h 30 min–9 h in mecillinam (M = 3 h 20 min) and 2 h–7 h in trimethoprim (M = 4 h 10 min). These results show that the T_lag_ algorithm can determine MIC of sensitive isolates as early as 3 h 30 min, based on the minimum T_lag_ of 1 h 30 min (**Figure 2G**). Again, these results reflect the heterogeneity among clinical isolates and illustrate the wide range of antibiotic exposure times necessary to generate a T_lag_. Taken together, the possibility to obtain MIC results of sensitive strains is valuable for clinical management, since a high MIC within the susceptible range can be associated with an adverse outcome in patients with Gram-negative infections (Falagas et al., 2012).

### Morphotyping of Clinical UPEC Isolates

**FIGURE 3:**
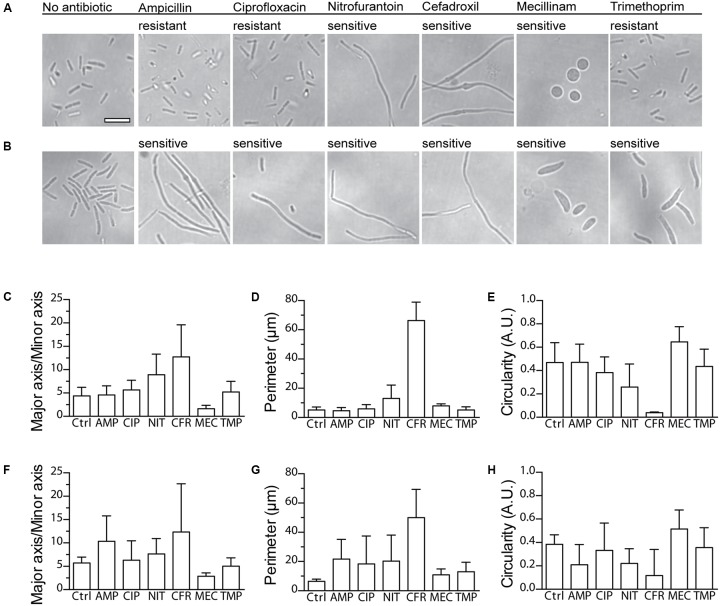
Imaging and morphotypic quantification of bacterial morphologies upon exposure to antibiotics. Phase contrast microscopy of the clinical UPEC isolates **(A)** ARD311 and **(B)** ARD318 in the nwAST shows the bacterial morphology after 3 h exposure to MIC (sensitive isolates) or breakpoint (resistant isolates) concentrations of each antibiotic. Scale bar = 5 μm, applies to all micrographs. Quantification of morphotypic parameters for strain **(C–E)** ARD311 and **(F–H)** ARD318 upon exposure to ampicillin (AMP), ciprofloxacin (CIP), nitrofurantoin (NIT), cefadroxil (CFR), mecillinam (MEC), and trimethoprim (TMP). Morphotypic parameters of major to minor axis, perimeter, and circularity were calculated based on image analysis from four technical replicates per antibiotic. The average and standard deviation is shown. Ctrl = no antibiotic added.

Absence of bacterial growth during the first hours of an AST is not necessarily due to inhibition by an antibiotic; it could also be due to an extended lag phase. This became evident in our experiments when a minority of resistant and susceptible isolates generated T_lag_ only after 4–5 h. Shortening of the incubation time less than that, although desirable, would thus entail the risk of misdiagnosis. In an attempt to solve this issue, we took advantage of the optical compatibility of the nwSlide, and the possibility this gives to perform microscopy of the nanocultures directly in the wells. It has been shown that antibiotics induce morphological changes in bacteria depending on their mode of action. Ampicillin, ciprofloxacin, nitrofurantoin, cefadroxil, and trimethoprim induce the formation of filaments while mecillinam induces the formation of ovoid cells (Spratt, 1975; Elliott et al., 1987; Mason et al., 1995; Chen et al., 2005; Ingham et al., 2006). We explored whether the morphological differences could be used to determine the susceptibility profile of bacteria. We selected one strain from the UPEC collection defined as clinically susceptible to all antibiotics in the nwAST (ARD318), and one resistant to ampicillin, ciprofloxacin, and trimethoprim (ARD311). After exposure of the isolates to the MIC or clinical breakpoint of each antibiotic for 3 h, we performed phase contrast imaging of bacteria in nanowells. In the absence of antibiotic, both strains appear as typical rod-shaped bacteria (**Figures 3A,B**). ARD311 maintained this morphology, even when exposed to antibiotics it showed resistance to (ampicillin, ciprofloxacin, trimethoprim). In contrast, the morphology deviated from the rod shape in both strains, when bacteria were exposed to antibiotics they were susceptible to. ARD311 became filamentous in the presence of nitrofurantoin and cefadroxil. Filamentation was also observed for ARD318 exposed to ampicillin, ciprofloxacin, nitrofurantoin, and cefadroxil. Under trimethoprim, filamentation and swelling were observed, whereas the expected ovoid and spherical morphology was seen under mecillinam.

Given the large number of nanowells, we expect that automation of the morphotype analysis could aid in reducing the workload and increase the precision of the AST. As a proof of concept, we analyzed the observed morphotypes for three different parameters: (i) the ratio between major and minor axis, (ii) perimeter, and (iii) circularity. As shown in **Figures 3C–H**, this three-parameter analysis efficiently discriminates rod-shaped, filamentous, swollen, or round morphotypes. Collectively, these results suggest that direct morphotyping on the nwSlide could potentially be applied after a short incubation to differentiate between susceptible and resistant strains. This added feature could complement T_lag_-based MIC determination in cases where growth is not detected within the first hours of incubation.

## Discussion

Clinical validation with 70 clinical UPEC isolates showed that the nwAST surpassed most of the FDA’s key performance criteria for AST systems (U.S. Department of Health and Human Services et al., 2009). The total CA between the nwAST and the reference disk diffusion assay was of 97.9% for all antibiotic-isolate combinations tested. This is higher than the minimum 90% required, which clearly demonstrates the accuracy of the nwAST. Importantly, no isolates were misidentified as sensitive instead of resistant, resulting in no VME. This type of error would have serious consequences for treatment as it could lead to the selection of an ineffective antibiotic therapy. The only discrepancies found was an overall ME of 2.6% because of a few sensitive strains misclassified as resistant. A likely explanation for most of these discrepancies resides in the manual coating of the slide, which can occasionally lead to batch variations. We foresee that automatization of the coating process will solve this issue and guarantee the quality of the assay.

Antibiotic susceptibility in agar plate methods and broth dilution is typically assessed between 18 and 22 h, when bacteria have entered stationary phase. The advantage of the nwAST over these methods is the ability to detect the time point of transition from lag to exponential phase using the T_lag_ algorithm. Based on T_lag_, the nwAST provides a median diagnostic time of 5 h 30 min for resistant isolates and 5 h 40 min for susceptible isolates. Diagnostic time can be shortened by 1 h, if optical recordings are performed every five instead of 10 min, as previously shown (Weibull et al., 2014). Moreover, application of automated T_lag_ analysis in real-time, would enable individualization of assay duration for each isolate tested. Overall, the nwAST shortens the time for diagnostics up to one third compared to current methods used in clinical practice.

The variability observed in the lengths of the lag phases reflects the natural heterogeneity amongst clinical strains. It is thus essential for any phenotypic AST that the assay time is not shorter than the strain’s lag phase. If so, a potentially resistant strain with a long lag phase could be misidentified as susceptible. This is often overlooked in studies aimed to develop novel ASTs. While others often restrict their study to the use of one or two antibiotics (Baltekin et al., 2017; Schoepp et al., 2017), the nwAST includes a panel of relevant antibiotics whose modes of action on bacterial physiology differ. In an effort to reduce the diagnostic time for isolates with long lag phase, we explored morphotyping as a complementary method. The glass bottom of the nwSlide makes it optically compatible with a range of microscopy techniques, and the small size of the wells facilitates location and surveillance of bacteria even when present in small numbers. In a proof of concept, we showed that susceptible bacteria deviate from the typical rod-shape morphology while resistant bacteria do not. Further investigation into the association of morphotype and susceptibility profile may provide us with a useful tool to complement T_lag_ analysis for faster AST results. Quantification of the shape parameters demonstrated that the morphotype analysis can be extended to differentiation between different species in mixed samples. This may become a highly valuable feature that enables simultaneous AST and species identification.

The nwAST is a quantitative method. Thanks to the large number of nanowells available on the slide, the nwAST incorporates antibiotic concentrations above and below the clinical breakpoints as well as technical replicates. This enables the determination of MICs not only for resistant, but also for susceptible strains, which is equally important for an optimal treatment of the patient. Furthermore, the use of defined antibiotic concentrations and optical recordings helps to eliminate the possible bias of manually determining the MIC by Etest^®^ (Mondino et al., 2003; Friedrich et al., 2009; Cantón et al., 2017). The T_lag_ analysis of the susceptible UPEC isolates establishes the minimum diagnostic time to determine a MIC at 3 h 30 min.

In addition to clinical UPEC isolates, we showed that other uropathogens such as *Klebsiella* spp*., Morganella morganii, Proteus mirabilis, Citrobacter koseri*, and *Enterobacter aerogenes* can be cultivated in the nanowells. This indicates that the nwAST can expand its scope beyond UPEC. The versatility of the slide allows for easy adaptation to a broader clinical setting, as the choice and number of antibiotics can be tailored to also include Gram-positive bacterial infections. An extra advantage is that the nwAST consumes 300-fold less antibiotics per well compared to its equivalent in standard microtiter plate assays, qualifying as a sustainable method for clinical practice.

Here, we have clinically validated the nwAST as an accurate and fast AST method. By applying the T_lag_ algorithm to nanocultures exposed to antibiotics, we were able to identify resistant clinical UPEC isolates as early as 3 h 40 min. The possibility of performing direct imaging of the isolates enables morphotyping of bacteria as an additional feature to enable rapid diagnosis. We anticipate that coupling a growth detection method with automated image analysis on the same device could boost the potential of the nwAST as a rapid and accurate clinical diagnostic tool. Ultimately, we envision that the nwAST will have impact beyond the immediate treatment of a patient. The potential of the nwAST as a tool for surveillance of pathogens in human and animal medicine, as well as in the environment can contribute to the ongoing global efforts to fight the spread of antimicrobial resistance.

## Author Contributions

HA, HA-S, AB, and AR-D conceived the idea of the nwAST device for clinical use. MV-G and HA performed all experiments. AB provided clinical guidance, collected, and pre-validated clinical isolates using standard clinical techniques. MV-G, HA, SL, AB, and AR-D analyzed the data. MV-G, HA, and AR-D wrote the manuscript with the contribution of AB, SL, and HA-S.

## Conflict of Interest Statement

The authors declare that the research was conducted in the absence of any commercial or financial relationships that could be construed as a potential conflict of interest.
