# Supplementary material for: Rapid Phenotypic Antibiotic Susceptibility Testing of Uropathogens Using Optical Signal Analysis on the Nanowell Slide
Source: Front Microbiol. 2018 Jul 10;9:1530. doi: 10.3389/fmicb.2018.01530 (PMC6048231; doi:10.3389/fmicb.2018.01530)
Supplement: Supplementary file 1 [file Data_Sheet_1.DOCX]

Supplementary Material

**Rapid Phenotypic Antibiotic Susceptibility Testing Of Uropathogens Using Optical Signal Analysis On The Nanowell Slide**

**Marta Veses-Garcia^1^, Haris Antypas^1^, Susanne Löffler^1^, Annelie Brauner^2^, Helene Andersson‑Svahn^3^, Agneta Richter-Dahlfors^1*^**

^1^Swedish Medical Nanoscience Center, Department of Neuroscience, Karolinska Institutet, Stockholm, Sweden.

^2^Department of Microbiology, Tumor and Cell Biology, Division of Clinical Microbiology, Karolinska Institutet and Karolinska University Hospital, Stockholm, Sweden.

^3^Division of Proteomics and Nanobiotechnology, Science for Life Laboratory, KTH-Royal Institute of Technology, Stockholm, Sweden.

**^*^Correspondence:** Agneta Richter-Dahlfors, agneta.richter.dahlfors@ki.se

**Supplementary Materials and Methods**

**Nanoscale cultures of UTI pathogens**

In order to become an efficient diagnostic tool, the nwAST must support the growth of diverse bacterial pathogens. To further demonstrate the versatility of the nanowell slide, we performed nanocultures of 6 typical UTI pathogens: *Klebsiella pneumonia*, *Klebsiella oxytoca, Proteus mirabilis, Morganella morganii, Enterobacter aerogenes* and *Citrobacter koseri*. Clinical isolates of each species were obtained from Karolinska University Hospital. A bacterial suspension of 5x10^5^ cfu/ml was used to inoculate the slides and they were incubated for 12 h at 37 °C. Absorbance recordings at 600 nm (OD_600_) were taken every 10 min. The OD_600_ recordings from 50 randomly selected nanocultures were plotted in heatmaps to easily visualize bacterial growth. Each row in the heatmaps represents bacterial growth in one nanoculture exemplified by the change of color from yellow to red.

**nwAST of *E.coli* ATCC 25922 reference strain**

To optimize the nwAST assay we performed 12 biological replicates using the reference strain *E.coli* ATCC 25922. A bacterial suspension of 5x10^5^ cfu/ml was used to inoculate each of the 12 slides containing antibiotics, which were incubated for 12 h at 37 °C with OD_600_ recorded every 10 min. For easy visualization of the growth data, OD_600_ from 336 individual nanocultures per antibiotic from all biological replicates were plotted in a heatmap. Each row represents bacterial growth in one nanoculture exemplified by the change of color from yellow to red. The MIC is defined as the first concentration where there is no change of color in at least 3 out of the 4 technical replicates.


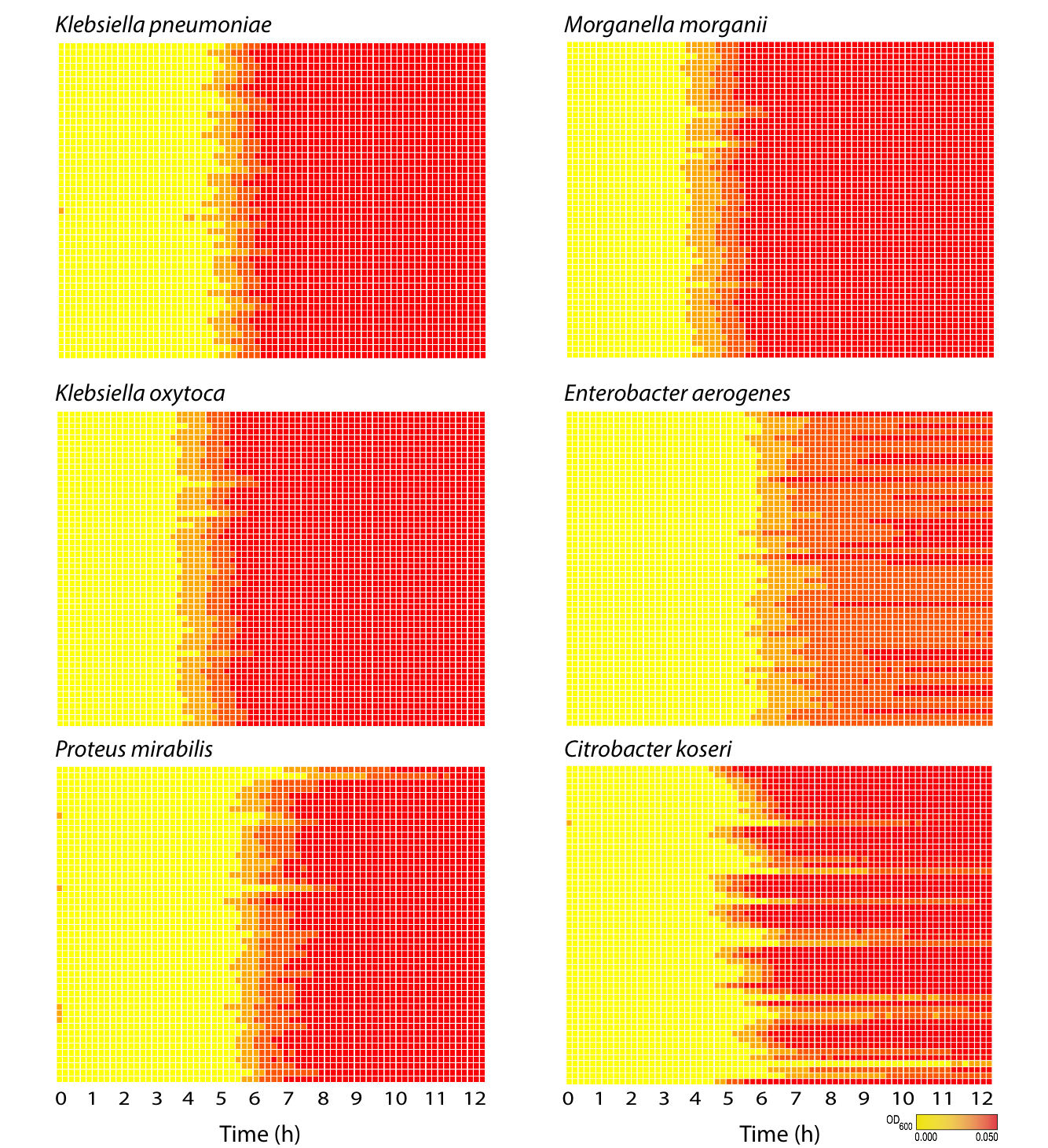


**Fig. S1. Nanoscale cultures of UTI pathogens.** Heatmaps representing the growth of 50 randomly selected nanocultures from the UTI pathogens *Klebsiella pneumonia*, *Klebsiella oxytoca, Proteus mirabilis, Morganella morganii, Enterobacter aerogenes* and *Citrobacter koseri*. Each of the 50 rows represent the growth in one nanowell and OD_600_ increase is illustrated by the colour change from yellow to red.


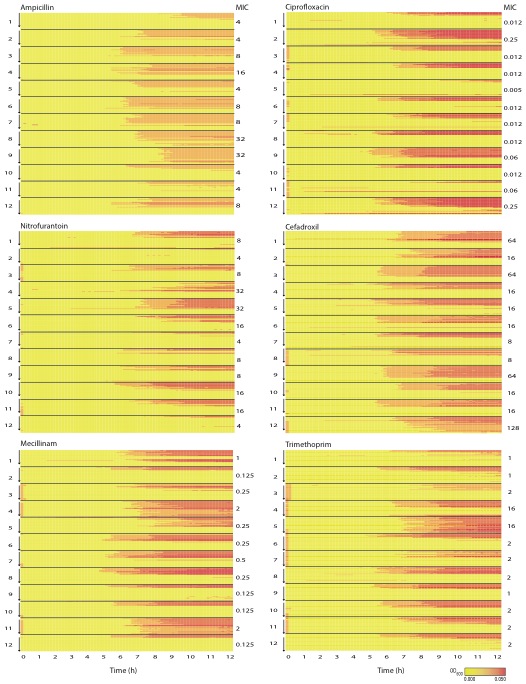


**Fig. S2**. **Heatmap analysis of 12 biological replicates performed with the reference strain *E.coli* ATCC 25922.** The replicate number is indicated on the left axis. Arrows indicate an increase of antibiotic concentration: ampicillin 0.25-16 µg/ml, ciprofloxacin 0.003- 0.25 µg/ml, nitrofurantoin 2-128 µg/ml, cefadroxil 2-128 µg/ml, mecillinam 0.03-2 µg/ml and trimethoprim 0.25-16 µg/ml. The MIC (µg/ml) determined in each biological replicate is indicated in the right axis.
